# Sources of Information as Determinants of Product and Process Innovation

**DOI:** 10.1371/journal.pone.0152743

**Published:** 2016-04-01

**Authors:** Jaime Gómez, Idana Salazar, Pilar Vargas

**Affiliations:** Departamento de Economía y Empresa, Universidad de La Rioja, Logroño, La Rioja, España; University of Reading, UNITED KINGDOM

## Abstract

In this paper we use a panel of manufacturing firms in Spain to examine the extent to which they use internal and external sources of information (customers, suppliers, competitors, consultants and universities) to generate product and process innovation. Our results show that, although internal sources are influential, external sources of information are key to achieve innovation performance. These results are in line with the open innovation literature because they show that firms that are opening up their innovation process and that use different information sources have a greater capacity to generate innovations. We also find that the importance of external sources of information varies depending on the type of innovation (product or process) considered. To generate process innovation, firms mainly rely on suppliers while, to generate product innovation, the main contribution is from customers. The potential simultaneity between product and process innovation is also taken into consideration. We find that the generation of both types of innovation is not independent.

## Introduction

The objective of this paper is to study the importance of different sources of information on product and process innovations. For years, the process of obtaining innovations was developed under the logic of closed innovation, where internal R&D investment was the most important factor of the innovation process [1,2]. The problem with this model is that, if a firm is too internally focused, it misses out on the contribution of external knowledge in its innovation activities. This fact caused a shift in the conception of how firms generate new ideas and bring them to the market, giving rise to the open innovation model. Chesbrough ([3]: 24) defines open innovation as *“a paradigm that assumes that firms can and should use external ideas as well as internal ideas*, *and internal and external paths to market*, *as firms look to advance their technology*”. This perspective redefines the limits between the firm and its environment in terms of innovation activities, making companies more porous and more likely to be embedded into networks of different actors [4,5].

The open innovation model highlights the role of a variety of useful external sources of information such as lead users, suppliers, rivals and universities among others [4,6]. Openness implies a commitment with external sources, but not a total dependence on them [7]. Attention to external sources does not diminish the relevance of internal knowledge [8], as firms that invest in internal sources of innovation are better able to recognize and evaluate external information [9]. Additionally, they are also more able to integrate and use their knowledge [9]. The evidence on complementarities between external and internal knowledge confirms this arguments (see, for example [10,11]).

Although open innovation is a rather new concept, it has received a significant amount of attention from both academics and firms [12]. The Connect+Develop strategy (http://www.pgconnectdevelop.com/) introduced by Procter and Gamble [13] or the Chorus model of Eli Lilly (http://www.choruspharma.com) are examples of the adoption of an open innovation model. Open innovation models have also produced substantial improvements in time and cost in R&D activities undertaken by pharmaceutical firms [13–15]. However, despite the growing body of knowledge, quantitative studies involving large samples are needed to understand the consequences of open innovation [12,16].

Rigby and Zook [17] describe the benefits of opening the innovation process to external knowledge flows. Firms that use external sources can broaden their knowledge base and access and integrate a greater variety of ideas to create new products and processes. They are also in a better position to face the challenges of increasing R&D risks and costs, shorter product life cycles and faster renewal [1,3,17,18]. However, as Laursen and Salter [4] have argued, an excessive reliance on external knowledge can be harmful. The reason is that an intensive use of external sources generates costs that must be taken into consideration. Some organizations over-search, spending too much time and effort looking for external sources [2], which may be detrimental in terms of innovation performance. Additionally, an excessive reliance on external information sources increases coordination and monitoring costs and could affect the building of knowledge stocks within the firm [1,19].

Previous studies have shown the effect of internal and external sources of information on the novelty of product innovation [4,20]. However, new products are not the only result of open innovation; service and process innovations are other important results of open innovation practices, either to create enhanced customer support or to support internal business efficiencies [21]. A smaller number of studies have centered on process innovation (see, for example [22]). Nevertheless we are not aware of any studies that analyze how different sources of information affect the development of both product and process innovation. Additionally, the literature often ignores the extent to which different kinds of innovation rely on different sources of knowledge. This study addresses these gaps by providing a comprehensive analysis of the sources of information that affect the generation of product and process innovation. Furthermore, the introduction of product innovation may cause the development of process innovations, and process innovation can stimulate the production of product innovations. Previous research (see, for example [23,24]) suggests the existence of complementarities between product and process innovation. For example Reichstein and Salter ([22]: 677) suggest that: *“the two types of innovation should be seen as “brothers” rather than “distant cousins*”. Analyzing these questions allows a deeper understanding of the innovation process at the firm level by identifying internal and external factors that affect the joint generation of the two types of innovation.

We use information from the Spanish Technological Innovation Panel (PITEC), (accessible from http://icono.fecyt.es/PITEC/Paginas/por_que.aspx), for 2005–2012. This data offers information on the innovation activities of a large sample of Spanish manufacturing firms, including the type of innovations obtained (product and process) and the sources of information used. Additionally, the data allows us to distinguish between three types of process innovations: innovations in manufacturing methods, in logistics and in supporting activities. We expect the sources of information used by the firm to be positively related to the introduction of both product and process innovation. At the same time, our hypothesis is that the pattern of influence will differ between product and process innovations and, in the case of the latter, between the three types identified. The methods of analysis are bivariate and multivariate probit models where the dependent variable is the generation of product or process innovation, which is explained by internal and external sources of information and several control variables.

## Materials and Methods

### Sample and data

The empirical analysis was carried out using the Spanish Technological Innovation Panel (PITEC). PITEC is sponsored by the *Fundación Española para la Ciencia y la Tecnología* (FECYT) and the COTEC Foundation and managed by the National Institute of Statistics. The dataset, the questionnaire and the description of each variable are available free of charge from: http://icono.fecyt.es/PITEC/Paginas/por_que.aspx.

This dataset has been used previously to study the influence of external and internal factors on the degree of innovation [25], the complementarity effect of R&D on firm productivity [26] and the relationship between R&D cooperation and environmental innovations [27].

This data set is important as it contains information on the introduction of product innovation and different types of process innovation. Furthermore, it includes information about the importance of different sources of information for the innovation process. The sources of information are divided into internal and external. The data set also allows controlling for firm and industry characteristics. Among the variables provided, we have data on the new products sold by the firm, the capital structure and the sector to which the firm belongs.

Although the information is available from 2003, some firms were incorporated into the data set in 2003 and others in 2004. In order to use a comparable set of firms, we use information for 2005–2012. The initial sample includes around 12,000 firms per year and 85,468 observations. The final sample was created after taking into account the following considerations. First, like Amara and Landry [20], we only use innovative firms belonging to the manufacturing sectors. Second, we exclude public firms and those that had undergone start-ups, mergers and closures. Third, we exclude firms that did not provide the necessary information to build our variables. As a result, our sample is composed of 34,964 observations. Table 1 displays a first approximation to the data set, showing a brief description of firms by size and sector over the period 2005–2012. We have split the sample into two sub-samples: large firms with 200 or more employees and small firms with fewer than 200 employees. Table 1 shows the composition of the final sample: approximately 82% are small firms and 18% large firms.

**Table 1:** Number of firms by size and industry.

Industry	Whole sample	> = 200 employees	<200 employees
1. Coke and refined petroleum products	0.05%	0.25%	0.00%
2. Food products, Beverages, and tobacco products	13.47%	19.20%	12.20%
3. Textiles	3.62%	1.51%	4.09%
4. Clothing	1.58%	2.11%	1.46%
5. Leather and related products	1.10%	0.38%	1.27%
6. Wood and cork	1.78%	1.46%	1.85%
7. Paper and paper products	1.96%	3.55%	1.60%
8. Printing and reproduction of recorded media	1.52%	0.94%	1.65%
9. Chemicals	10.25%	7.30%	10.91%
10.Pharmaceutical products	2.74%	6.43%	1.92%
11. Rubber and plastic products	6.63%	4.95%	7.00%
12. Other non-metallic mineral products	5.72%	7.20%	5.39%
13. Basic metals	2.88%	6.32%	2.12%
14. Fabricated metal products, except machinery and equipment	10.69%	6.90%	11.53%
15. Computer, electronic and optical products	4.95%	3.08%	5.37%
16. Electrical equipment	4.80%	4.31%	4.91%
17. Machinery and equipment	12.49%	5.88%	13.96%
18. Motor vehicles, trailers and semi-trailers	4.72%	11.50%	3.22%
19. Building of ships and boats	0.36%	0.09%	0.42%
20. Air and spacecraft and related machinery	0.37%	0.94%	0.24%
21. Other transport equipment	0.52%	0.93%	0.43%
22. Furniture	3.40%	1.79%	3.75%
23. Other manufacturing	2.58%	1.59%	2.80%
24. Repair and installation of machinery and equipment	1.83%	1.35%	1.94%
**Total manufacturing**	**100%**	**100%**	**100%**

### Variable description and measurement

#### Dependent variables

The two dependent variables capture whether the firm has generated product and process innovations. The Spanish Technological Innovation Panel was designed according to the guidelines of the Oslo Manual [28]. Therefore, the definitions of product and process innovations are consistent with these guidelines.

*Product innovation*. The introduction of product innovation is measured through a dummy variable that takes the value 1 when the firm introduced new or significantly improved goods (the simple resale of new goods and changes of a solely aesthetic nature are not included) in the previous two years, and 0 otherwise.

*Process innovation*. The introduction of process innovations is measured through a dummy variable that takes the value 1 when the firm introduced a new or significantly improved production process, distribution method, or supporting activity in the previous two years, and 0 otherwise. In order to find out whether the effect of the sources of information varies depending on the type of process innovation, we consider three different types of process innovation, namely, innovation in manufacturing methods, in logistics and in supporting activities. Accordingly, we define three dependent variables that take the value 1 when the firm introduced new or significantly improved methods of manufacturing or producing goods (*Manufacturing methods innovation*), logistics, delivery or distribution methods for inputs or goods (*Logistic innovation*) or supporting activities for processes, such as maintenance systems or operations for purchasing, accounting or computing (*Supporting activities innovation*), and 0 otherwise.

#### Independent variables

The database contains data about the importance of internal and external sources of information. The PITEC considers 10 different sources: one internal and nine external. We only selected external sources from market and scientific agents [29,30]. Although we also have information on the use of other external sources, i.e. conferences and professional associations, we do not use them in order not to incur double counting.

Specifically, we consider the importance of each of the following: (1) within the enterprise or enterprise group (*Internal*), (2) suppliers of equipment, materials, components or software (*Suppliers*), (3) customers (*Customers*), (4) competitors or other enterprises in the sector (*Competitors*), (5) consultants, commercial labs or private R&D institutes (*Consultants*) and (6) universities or other higher education institutions (*Universities*). Firms are asked to rate the importance of each source on a four-point scale (none/not used, low, medium and high). We recodified these variables to obtain measures ranging between 0 (none) and 1 (high importance).

#### Control variables

The literature has suggested a number of firm and industry variables that could affect the likelihood of innovation. First, larger firms are usually more likely to introduce product and process innovations, given that they have more resources. We include the sales of the firm (in thousand euros) (*Size*) as a proxy for this variable [31]. Second, according to Cohen and Levintal [9] firms with higher levels of absorptive capacity are more likely to innovate. To control for this effect, we include the firm’s innovation intensity. Following Cassiman and Veugelers [10], this variable is calculated as the ratio of the total innovation investments to sales (*Innovation intensity*). Third, firms exposed to international markets are more likely to innovate [10]. Therefore, we expect a positive relationship between a firm’s export propensity (*Export propensity*) and the introduction of product and process innovations. This variable is measured through the ratio of exports to sales in a given year [32]. The presence of foreign capital could also affect the decision to innovate in a positive way. For this reason, we include a dummy variable (*Foreign capital*) that takes the value 1 when the participation of foreign investors in the firm’s capital is higher than 50%, and 0 otherwise [23].

When analyzing the factors affecting the decision to innovate, one important element to take into account is technological opportunity [33]. Firms belonging to high intensity sectors have greater access to knowledge and technological progress and, therefore, are more likely to innovate [34]. To control for this effect, we include a dummy variable (*High intensity sector*) that takes the value 1 when the firm belongs to a high intensity sector, and 0 otherwise. We have used the OECD [35] classification of manufacturing industries to form the high and the low intensity groups.

Finally, to control for time effects we include a set of time dummies that take a value of 1 for each of the years (2005–2012) considered.

### Model and methodology

The focus of this paper is the estimation of an innovation equation. We have five innovation outcomes. We know whether the firm has obtained *product* or *process* innovations. We also know the type of process innovation obtained: *manufacturing methods*, *logistic* and *supporting activities*.

The innovation equation follows the form:

$$I_{i,t}=f\left(SI_{i,t-1},X_{i,t-1}\right)$$ (1)

where I_it_ is the innovation outcome of interest, SI_it-1_ are the sources of information used by the firm and X_it-1_ the control variables capturing firm, industry and time effects.

Following Blundell, Griffith and Van Reenen [36], eq (1) can be derived as the outcome of a firm’s optimal search rule for innovation. The model considers that the search process generates innovation in future periods and that is why all independent variables are lagged. Previous papers (see, for example, [24]) have also used a single lag to capture the dynamic component of the model.

As we are interested in the effect of information sources on the innovation outcome, our empirical model takes the following form:

$$Innovation\;outcome_{i,t}^{*}=\alpha_{0}Internal_{i,\;t-1}+\alpha_{1}Suppliers_{i,\;t-1}+\alpha_{2}Competitors_{i,\;t-1}+\alpha_{3}Customers_{i,\;t-1}+\alpha_{4}Consultants_{i,\;t-1}+\alpha_{6}Universities_{i,\;t-1}+CV_{,\;t-1}+\varepsilon_{i,t}$$ (2)

where *CV* stands for control variables, namely, *High intensity*, *Export intensity*, *Foreign capital*, *R&D intensity*, *Size and Temporal dummies*, and *ε*_*i*,*t*_ and *ϑ*_*i*,*t*_ are the error terms. We expect the sources of information and all the control variables to have a positive impact on innovation, except for the *Temporal dummies*, whose effect is indeterminate.

One important issue that has to be considered is that the continuous latent variable, innovation outcome of firm *i* at time *t*, is not observed. We only observe whether firm *i* has or has not generated a given type of innovation, *Innovation outcome*_i,t._ The latent variable is related to the observed dependent variable such that [36]:

$$Innovation\;outcome_{i,t}=\{\begin{array}{c} 1\;if\;\;Innovation\;outcome_{i,t}^{*}\;>0\\ 0\;if\;\;Innovation\;outcome_{i,t}^{*}\leq 0 \end{array}$$ (3)

Due to the dichotomous nature of our dependent variables, we use the probit model as the base of all our estimations [37]. It is important to bear in mind that it is likely that the error terms of the different innovation outcomes are correlated. The correlation may be due to product innovations that involve the modification of the production process or to process innovations that result in the introduction of product innovations. However, it could also be the result of common factors affecting the probability of introducing these innovations and that the model does not control for. If this correlation is not considered, the estimated parameters could be biased and inconsistent [38]. To solve this problem, in the analysis of product and process innovation, we estimate a bivariate probit model. As we also distinguish between three types of process innovation (manufacturing methods, logistic and supporting activities), a natural extension of the probit model, the multivariate probit model proposed by Cappellari and Jenkins [39], is also applied. In both models, we cluster the errors by firm to control for the fact that observations are correlated within firms. All the models are estimated using Stata 13.

## Results and Discussion

The descriptive analysis and correlation matrix of the variables used are displayed in Table 2. This table first provides important information about the frequency of product and process innovations. Approximately 68% of the cases considered introduce product and process innovations. These figures also show that, of all the types of process innovation considered, manufacturing methods innovation is the most frequent (56%), while innovation in logistics is the least frequent (16%). Second, the data reveals the importance of the different sources of innovation used by the firms. Internal sources of information are the most important (78%). Among external sources, suppliers and customers present similar frequencies (with percentages of 52% and 54%, respectively). Universities are the source of information least frequently used by firms in the sample (24%). Third, the correlations between the explanatory variables are low, although the highest correlations are found between the different sources of information considered.

**Table 2:** Descriptive analysis and correlation matrix (n = 29,510).

	Mean	S.D.		(1)	(2)	(3)	(4)	(5)	(6)
**Product innovation (1)**	0.683	0.465		1					
**Process innovation (2)**	0.679	0.467		0.147	1				
**Manuf. methods innovation (3)**	0.560	0.496		0.174	0.775	1			
**Logistic innovation (4)**	0.156	0.363		0.124	0.296	0.213	1		
**Supporting activities (5)**	0.335	0.472		0.141	0.487	0.190	0.361	1	
**Internal (6)**	0.777	0.309		0.218	0.128	0.142	0.097	0.098	1
**Suppliers (7)**	0.522	0.348		0.128	0.172	0.149	0.116	0.152	0.279
**Competitors (8)**	0.544	0.372		0.256	0.090	0.104	0.096	0.121	0.336
**Customer (9)**	0.405	0.343		0.200	0.089	0.096	0.099	0.112	0.243
**Consultants (10)**	0.329	0.337		0.102	0.115	0.103	0.106	0.126	0.175
**Universities (11)**	0.241	0.319		0.112	0.086	0.096	0.104	0.098	0.173
**High intensity sector (12)**	0.439	0.496		0.144	-0.067	-0.050	0.010	0.013	0.108
**Export propensity (13)**	0.272	0.299		0.079	0.064	0.089	0.032	0.029	0.092
**Foreign capital (14)**	0.074	0.262		0.020	0.033	0.034	0.050	0.007	0.034
**R&D intensity (15)**	0.041	0.084		0.102	0.013	0.023	-0.010	0.019	0.096
**Size (16)**	52125.9	317563.3		0.034	0.051	0.046	0.096	0.059	0.043
	**(7)**	**(8)**	**(9)**	**(10)**	**(11)**	**(12)**	**(13)**	**(14)**	**(15)**
**Suppliers (7)**	1								
**Competitors (8)**	0.370	1							
**Customer (9)**	0.364	0.582	1						
**Consultants (10)**	0.300	0.270	0.338	1					
**Universities (11)**	0.194	0.229	0.237	0.432	1				
**High intensity sector (12)**	-0.001	0.134	0.102	-0.017	0.085	1			
**Export propensity (13)**	0.017	0.092	0.072	0.053	0.082	0.128	1		
**Foreign capital (14)**	-0.004	-0.008	-0.012	0.006	0.014	0.072	0.162	1	
**R&D intensity (15)**	0.044	0.122	0.082	0.065	0.130	0.119	-0.019	-0.061	1
**Size (16)**	0.044	0.003	0.029	0.043	0.064	0.009	0.053	0.123	-0.042

**Fig 1:**
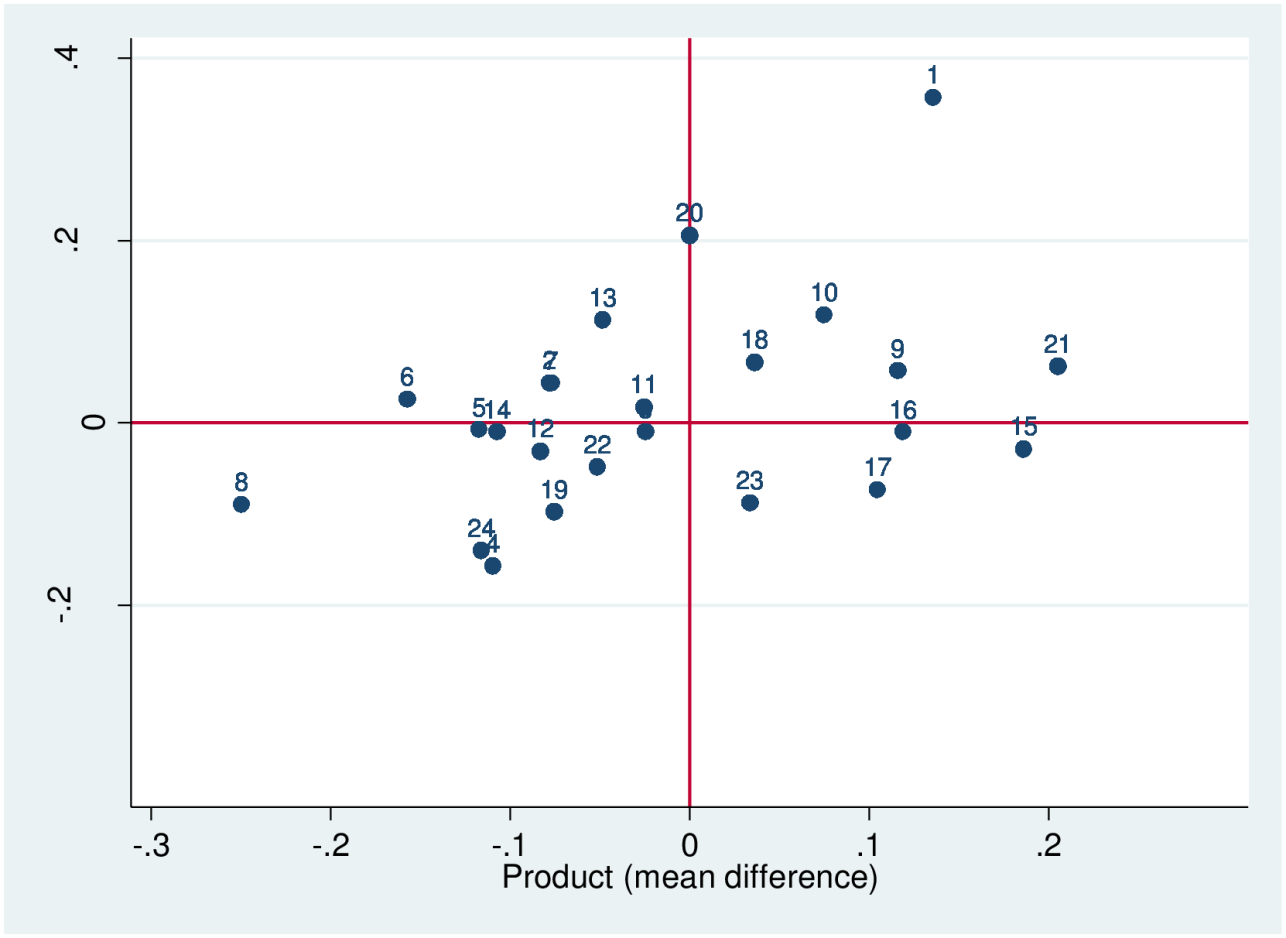
Differences in product and process innovation between industries.

Fig 1 shows information on product and process innovations by industry. It depicts the proportion of firms that have innovated in each of the industries considered (see Table 1 for a description of these industries). To plot the position of each industry, we calculated the proportion of product and process innovators in each industry and subtracted the mean proportion of all the industries. Therefore, the lines going through 0 on the x-axis and the y-axis represent, respectively, the average of the proportion of product and process innovators in manufacturing. Industries to the right of the x axis are above the mean in terms of the percentage of product innovators, whereas industries to the left of the x axis are below the mean. Similarly, industries at the top of the figure have a larger proportion of process innovators than the overall average, and industries below the red line are below average proportion of process innovators. Fig 1 not only reveals industry differences in product and process innovations, but also whether the two types of innovation tend to take place simultaneously.

Fig 1 shows that some industries with the highest levels of product innovation also have high levels of product innovation. This is the case of the petroleum industry, the pharmaceutical industry and the motor vehicles industry. However, there are also some industries with high rates of process innovation and low rates of process innovation like basic metals, rubber and plastic, wood and cork, paper and food. Finally, there are certain industries with high rates of product innovation and low rates of process innovations—machinery and equipment and computer, electronic and optical products. These results are consistent with Reichstein and Salter [23] and generally suggest that, in industries with high technological opportunity, there is a greater probability of obtaining both product and process innovation.

Table 3 shows the estimation of a bivariate and a multivariate probit model using the 29,510 observations available. The number of observations available for the analysis (34,964) is higher than the number of observations in the estimation (29,510). We lose observations as a consequence of the lag of the independent variables. Columns 1 and 2 present the determinants of product and process innovation. Columns 3 to 6 estimate the model with the same sample, but distinguishing between different types of process innovation. The table also shows the result of estimating the correlation between the different types of innovations (the rhos at the bottom). All the models are globally significant, as revealed by the Wald tests. Similarly, the observation of the variance inflation factors (VIFs) indicates that there are no multicollinearity problems because they show values below the usual thresholds.

**Table 3:** Estimates of the decision to introduce product and process innovation.

	Bivariate probit		Multivariate probit			
	Product innovation _t_	Process innovation _t_	Product innovation _t_	Manufacturing methods innovation _t_	Logistic innovation _t_	Supporting activities innovation _t_
**Internal**_**t-1**_	0.535*** (14.37)	0.329*** (9.11)	0.534*** (14.37)	0.390*** (10.87)	0.285*** (6.02)	0.159*** (4.15)
**Suppliers**_**t-1**_	0.022 (0.58)	0.502*** (14.04)	0.023 (0.61)	0.355*** (10.15)	0.310*** (7.18)	0.391*** (10.76)
**Customers**_t-1_	0.550*** (13.83)	-0.002 (-0.05)	0.550*** (13.86)	0.056 (1.46)	0.081*** (1.71)	0.138*** (3.47)
**Competitors**_t-1_	0.243*** (5.69)	0.024 (0.56)	0.244*** (5.70)	0.049 (1.19)	0.118** (2.38)	0.060 (1.39)
**Consultants**_t-1_	0.022 (0.56)	0.190*** (4.76)	0.023 (0.58)	0.108*** (2.82)	0.186*** (4.06)	0.235*** (6.03)
**Universities**_t-1_	0.107** (2.40)	0.115*** (2.60)	0.110** (2.49)	0.174*** (4.19)	0.221*** (4.44)	0.116*** (2.75)
**High intensity sector**_t-1_	0.272*** (9.40)	-0.244*** (-8.63)	0.272*** (9.42)	-0.208*** (-7.59)	-0.037 (-1.11)	-0.003 (-0.12)
**Export propensity**_t-1_	0.209*** (4.48)	0.296*** (6.60)	0.208*** (4.47)	0.376*** (8.70)	0.051 (0.98)	0.082* (1.90)
**Foreign capital**_**t-1**_	0.072 (1.50)	0.177*** (3.79)	0.070 (1.47)	0.164*** (3.65)	0.208*** (4.16)	0.034 (0.74)
**Innovation intensity**_t-1_	1.020*** (6.00)	0.116 (0.84)	1.02*** (6.05)	0.204 (1.54)	-0.519*** (-2.78)	-0.006 (-0.05)
**Size**_t-1_	0.000 (1.40)	0.000** (2.10)	0.000 (1.52)	0.000* (1.82)	0.000*** (2.59)	0.000*** (2.72)
**Time dummies**	Included	Included	Included	Included	Included	Included
**Constant**	-0.892*** (-22.67)	-0.495*** (-12.98)	-0.882*** (22.46)	-0.769*** (20.28)	-1.776*** (34.04)	-1.232*** (29.82)
**VIF (max/mean)**	2.12/1.51					
**Wald test**	3,198.20		3,495.59			
**Observations**	29,510		29,510			
**Rho**_**Product, process**_	0.148***		**Rho**_**Product, manuf.**_	0.186***	**RhoManuf., logistic**	0.317**
			**Rho**_**Product, logistic**_	0.159***	**RhoManuf., support**	0.229***
			**RhoProduct, support**	0.146***	**RhoLogistic, support**	0.493***
**LR test de Rhoi,j = 0 ∀ j≠k**	232.508		**4,280.18**			

***, **, *: Variable statistically significant at 1%, 5% or 10%, respectively. T-ratios in parentheses

Regarding the influence of information sources on product innovation, column 1 reveals the positive and significant impact of four of the six sources considered, as expected. Information from customers ($\hat{\beta }$ = 0.550 p<0.01) and from internal sources ($\hat{\beta }$ = 0.535; p<0.01) are the ones with the highest impact on the likelihood of obtaining product innovation. Information from competitors ($\hat{\beta }$ = 0.243; p<0.01) and universities ($\hat{\beta }$ = 0.107; p<0.05) are the ones with the lowest impact on new products. Information from suppliers ($\hat{\beta }$ = 0.022; p>0.1) and consultants ($\hat{\beta }$ = 0.022; p>0.1) does not significantly affect product innovations.

Of the effects of the different sources (of information) on process innovation, the most important is that obtained from suppliers ($\hat{\beta }$ = 0.502; p<0.01), followed by internal information ($\hat{\beta }$ = 0.329; p<0.01) and by that coming from consultants ($\hat{\beta }$ = 0.190; p<0.01) and universities ($\hat{\beta }$ = 0.115; p<0.01). Information from customers and competitors does not have a significant influence ($\hat{\beta }$ = -0.002; p>0.10 and $\hat{\beta }$ = -0.024; p>0.10 respectively). The estimates also suggest that product and process innovation are sometimes produced together, given the positive and significant correlation between the two (**Rho**_**Product, process**_ = 0.148; p<0.01). This correlation, however, seems low and, together with the results just described, suggests that there are differences in the production of the two types of innovation (we further explore this issue in the next section).

Columns 3 to 6 present the results of estimating a multivariate probit model that shows the effect of information sources on product innovation and on the different types of process innovation. They confirm the pattern just described for product innovations: the change in the values of the estimated coefficients is negligible, with customers ($\hat{\beta }$ = 0.550; p<0.01) and the firm (internal) ($\hat{\beta }$ = 0.534; p<0.01) being the most important sources. However, they also show some differences when the types of process innovations are considered. Among the commonalities, it is important to highlight that the most important source of information for process innovation are suppliers. A comparison of the coefficient accompanying this variable in the three types of process innovations shows that the differences are not significant (χ^2^ = 3.24; p>0.1). The impact of universities on process innovation is also similar, given that the Wald test (χ^2^ = 4.37; p>0.1) does not show differences between coefficients.

If we focus on the differences, although internal sources appear among the three most important for the all the types of process innovations, the results show statistically significant differences (χ^2^ = 22.46; p<0.001). This is also the case with consultants (χ^2^ = 6.52; p<0.05), whose importance is the highest in supporting activities innovation ($\hat{\beta }$ = 0.235; p<0.01) and the lowest in the case of manufacturing methods innovation ($\hat{\beta }$ = 0.108; p<0.01). Finally, some of the sources considered are non-significant for certain types of process innovation: customers, in the case of manufacturing innovations ($\hat{\beta }$ = 0.056; p>0.1), and competitors, in the cases of manufacturing ($\hat{\beta }$ = 0.049; p>0.1) and supporting activities innovation ($\hat{\beta }$ = 0.060; p>0.1). Overall, these results suggest that information sources affect process innovations differently, depending on their type.

It is also important to note that manufacturing methods innovation is the type of process innovation more likely to take place together with product innovation (Rho_Product, manuf._ = 0.186; p<0.01). The correlations between the different types of process innovation also suggest that they are frequently tied. Process innovation in the areas of supporting activities and logistics have a higher correlation (Rho_Logistic, support_ = 0.493; p<0.01), followed by supporting activities and manufacturing methods innovation (Rho_Manuf., logistic_ = 0.317; p<0.01).

Regarding the effect of control variables, the results show that the sector to which the firm belongs also has an effect on product and process innovation. Firms in high technology sectors are more likely to generate product innovations ($\hat{\beta }$ = 0.272; p<0.01). However, these firms are less likely to introduce process innovations ($\hat{\beta }$ = -0.244; p<0.01). The analysis of the different types of process innovation shows that this negative impact is driven by the negative and significant impact of this variable on manufacturing methods innovation ($\hat{\beta }$ = -0.208; p<0.01). The results also reveal that firms with high export propensity are more innovative in terms of product ($\hat{\beta }$ = 0.209; p<0.01) and process innovation ($\hat{\beta }$ = 0.296; p<0.01). The only exception is logistic innovation, where we cannot reject the null hypothesis ($\hat{\beta }$ = 0.051; p>0.1). Innovation intensity is positively related to product innovation ($\hat{\beta }$ = 1.020; p<0.01) and non-significant for process innovation. However, in the case of process innovations, we find that there is a negative association with innovation in logistics ($\hat{\beta }$ = -0.519; p<0.01). Finally, the explanation of product and process innovations is also different when size and foreign capital are considered. Both variables have a positive effect on process innovation, while it is non-significant for product innovations. These results are in line with previous research (see, for example, [23]) and seem to suggest that large firms and firms with foreign capital have access to the complementary resources needed to appropriate the value from process innovations [40]. These findings are consistent with the literature on process technology adoption and the role of the two variables in shaping the adoption behavior of firms [41,42].

Finally, some papers argue that past innovation activities generate expertise about how to perform innovative activities [43,44]. To test this idea, we built a measure of innovation experience based on the number of years a firm has obtained product or process innovation in the past. The results (not shown) reveal that previous experience has a positive impact on all the types of innovation.

### Further analyses

The results presented in the previous sections suggest that product and process innovations could be related. However, the significant correlation between the different innovation outcomes could be due to complementarities or to the existence of unobserved heterogeneity. Furthermore, innovation activities may be persistent. To further explore these ideas, we performed additional analysis. More precisely, we estimated a dynamic probit specification that controls for the initial conditions problem (see [45,46]) To deal with heterogeneity we use Wooldridge’s [47] approach, which proposes that unobserved heterogeneity depends on the initial value of the dependent variable (*Initial conditions*_*0*_) and the mean of the exogenous variables. Given the lack of variation of the exogenous explanatory variables over time, we have considered a more restricted approach where unobserved heterogeneity is assumed to be correlated only with the initial value of the dependent variable [48]. To capture complementarities between product and process innovation, the product (process) innovation equation incorporates the lagged value of process (product) innovation.

Table 4 shows the results of a bivariate (multivariate) dynamic probit in which heterogeneity is controlled for in the way described above. The results suggest that the probability of obtaining a product (process) innovation in t depends on whether or not the firm has generated a product (process) in the past because the coefficient for product (process) innovation in t-1 is positive and significant. This result is similar to those of Peters [45] and Mañez et al. [46]. Further, our estimates also show that obtaining a process innovation in *t-1* increases the probability of obtaining product innovations in *t*. Similarly, achieving product innovations in *t-1* also increases the probability of obtaining innovations in logistics and in supporting activities. These results suggest a complementarity relationship between product and process innovation. This conclusion is further reinforced by the correlations between product and process innovations. They maintain their positive and significant coefficients (see the *Rho* correlations at the bottom of Table 4). Note that, in this case, the models control for unobserved heterogeneity.

**Table 4:** Estimates of the decision to introduce product and process innovation (dynamics and unobserved heterogeneity).

	Bivariate probit		Multivariate probit			
	Product innovation _t_	Process innovation _t_	Product innovation _t_	Manufacturing methods innovation _t_	Logistic innovation _t_	Supporting activities innovation _t_
**Product innovation**_**t-1**_	2.140*** (52.10)	0.049 (1.39)	2.141*** (52.23)	0.046 (1.41)	0.108*** (2.59)	0.072** (2.07)
**Process innovation**_**t-1**_	0.075** (2.17)	2.144*** (58.15)	0.059* (1.71)			
**Manufacturingmethod**_**t-1**_				1.942*** (62.25)		
**Logistic innovation**_**t-1**_					1.991*** (51.43)	
**Supporting activity**_**t-1**_						1.855*** (61.58)
**Internal**_**t-1**_	0.219*** (4.01)	0.137*** (2.58)	0.220*** (4.04)	0.131** (2.57)	0.200*** (3.03)	0.079 (1.46)
**Suppliers**_**t-1**_	0.091* (1.77)	0.267*** (5.69)	0.095* (1.85)	0.219*** (4.99)	0.152*** (2.80)	0.168*** (3.71)
**Customers**_**t-1**_	0.248*** (4.88)	0.075 (1.46)	0.249*** (4.91)	0.084* (1.77)	0.070 (1.25)	0.089* (1.89)
**Competitors**_**t-1**_	0.156*** (2.82)	-0.106** (1.98)	0.156*** (2.83)	-0.079* (1.65)	-0.029 (0.51)	-0.012 (0.23)
**Consultants**_**t-1**_	0.033 (0.65)	0.098** (1.97)	0.033 (0.66)	0.065 (1.43)	0.128** (2.36)	0.093** (2.01)
**Universities**_**t-1**_	0.019 (0.38)	0.080 (1.59)	0.019 (0.38)	0.095** (2.07)	0.166*** (3.03)	0.137*** (3.01)
**High intensity sector**_t-1_	0.111*** (3.65)	-0.142*** (4.90)	0.110*** (3.62)	-0.117*** (4.34)	-0.023 (0.69)	-0.023 (0.82)
**Export propensity**_**t-1**_	0.093* (1.87)	0.211*** (4.54)	0.093* (1.86)	0.254*** (5.82)	0.007 (0.14)	-0.002 (0.04)
**Foreign capital**_**t-1**_	0.077 (1.51)	0.075 (1.59)	0.078 (1.53)	0.085* (1.89)	0.125** (2.49)	0.048 (1.02)
**Innovation intensity**_t-1_	0.720*** (2.75)	-0.209 (1.12)	0.720*** (2.75)	-0.127 (0.70)	-0.431** (1.99)	0.012 (0.06)
**Size**_t-1_	0.000*** (2.59)	0.000* (1.84)	0.000** (2.52)	0.000*** (2.64)	0.000 (0.90)	0.000 (1.05)
**Initial conditions**_**0**_	0.211*** (5.48)	0.120*** (3.40)	0.211*** (5.51)	0.155*** (5.19)	0.302*** (7.77)	0.146*** (5.00)
**Time dummies**	Included	Included	Included	Included	Included	Included
**Constant**	-2.008*** (29.09)	-1.739*** (26.78)	-2.000*** (29.00)	-1.683*** (28.17)	-2.286*** (29.07)	-1.858*** (30.29)
**Wald test**	8875.78		14159.06			
**Observations**	14736		14736			
**Rho**_**Product, process**_	.110***		**Rho**_**Product, manuf.**_	0.130***	**Rho**_**Manuf., logistic**_	0.261***
			**Rho**_**Product, logistic**_	0.099***	**Rho**_**Manuf., support**_	0.141***
			**Rho**_**Product, support**_	0.094***	**Rho**_**Logistic, support**_	0.362***
**LR test deRhoi,j = 0 ∀ j≠k**	33.143***		753.791***			

***, **, *: Variable statistically significant at 1%,5% or 10%, respectively. T-ratios in parentheses

Additionally, we explored whether there are different patterns in the use of information sources depending on the level of technological intensity. We used the OECD industry aggregation [35] in order to distinguish between high and low technology sectors. This exercise showed important differences regarding the importance of internal sources. Specifically, the results show that internal sources are significant for obtaining process innovation in high technology sectors. However, in low technology sectors, internal sources are important for obtaining product innovations.

## Conclusions

In this paper, we have studied the importance of different sources of information in product and process innovation. The results lead us to two conclusions. First, they show that the six sources considered (internal, suppliers, customers, competitors, consultants and universities) play a role in producing innovation. These results are in line with previous papers that find that innovations are developed by using knowledge from a diverse set of internal and external sources of information (see, for example [20]) and not just from that generated by R&D investments. Additionally, we have shown that the influence of each source is different depending on the type of innovation. To obtain product innovations, firms mainly rely on customers and on internal sources, although information from competitors and universities is also important. To obtain process innovations, internal sources and suppliers are the main contributors, followed by consultants and universities. This means that only internal information and information from universities are important in both types of innovation. These differences are also found in the production of the three types of process innovations identified in our study.

Second, our findings reveal that there are complementarities between innovation types. These complementarities are small between product and process innovations and mainly involve the three types of process innovations. In the case of the latter, the relationship between logistic and supporting activities innovation is stronger than in the two combinations that include manufacturing methods innovation (manufacturing-logistic and manufacturing-supporting). Importantly, we detect complementarities even when controlling for unobserved heterogeneity.

Our results also have implications for our understanding of how innovation is produced and studied. They suggest the need to separate product and process innovations if they are to be studied effectively. This adds to recent evidence that shows that the influence of the various factors involved in the propensity to engage in product and process innovations is different. For example, Berchicci, Tucci and Zazzara [49] find that industrial downturns have a different impact on the product and process innovations of Italian manufacturing firms. We also find that the development of process innovations has distinguishing features, depending on their type. For example, internal sources are more important for manufacturing methods innovation than for supporting activities innovation.

Our paper also has implications in terms of the selection of a firm’s innovation model. The high speed of technological change has produced a shift towards open models of innovation in some firms. These models assume that even large firms with good resource endowments need information from external sources to produce innovation [3]. Our results confirm the importance of external sources of information, but they also highlight the importance of internal sources, especially for product innovations.

An open innovation model allows firms not only to obtain higher innovation performance but also to capture value [3]. For example, the R&D productivity of Procter and Gamble with the Connect and Develop strategy increased by nearly 60% [50]. Similarly, Roper et al. [51] found evidence of a direct link between the use of knowledge from external sources and business growth and productivity. In our sample, firms stating that both internal and external sources are important or very important obtain (statistically significant) higher levels of labor productivity (sales per employee) than rivals that do not.

In terms of policy prescriptions, these findings suggest that policies that encourage the links between firms and market and scientific sources of information should be promoted because they positively affect innovation performance. These policies should take into account that a varied mix of sources of information is beneficial to innovation performance. However, they should also consider the different nature and impact of the sources of information on product and process innovation in order to obtain the desired outcome. Keeping or providing incentives to investments in innovation (R&D, for example) would also benefit product innovation.

This study has limitations. First, the database contains information on a large number of firms and variables. However, it was not designed to evaluate the role of information sources on innovation performance. More information on the type of innovation outcome obtained would be interesting. For example, the distinction between product innovations with different degrees of novelty seems an interesting extension. Similarly, the data used in the empirical analysis consider the firm as the unit of analysis. However, this does not allow for a precise evaluation of how specific pieces of information are used. As firms are likely to organize their innovation efforts into projects, information at this level could provide a more detailed assessment. A second limitation is that our study refers to only one country, Spain. This means that the influence of certain environmental variables (institutional factors, for example) could condition the relationships identified.

These limitations open new opportunities for research. Researchers could examine the impact of information sources on the degree of novelty of innovations. They could also collect detailed information on how the fluxes of internal and external information are used in the innovation process. Additionally, the study could be performed using a sample of different countries to capture the consequences of different institutional and economic contexts.
